# The application of machine learning to imaging in hematological oncology: A scoping review

**DOI:** 10.3389/fonc.2022.1080988

**Published:** 2022-12-19

**Authors:** Stylianos Kotsyfakis, Evangelia Iliaki-Giannakoudaki, Antonios Anagnostopoulos, Eleni Papadokostaki, Konstantinos Giannakoudakis, Michail Goumenakis, Michail Kotsyfakis

**Affiliations:** ^1^ Diagnostic Center ‘Ierapetra Diagnosis’, Ierapetra, Greece; ^2^ General Hospital of Heraklion Venizeleio-Pananeio, Heraklion, Greece; ^3^ Agios Savvas Oncology Hospital of Athens, Athens, Greece; ^4^ ‘Apollonion’ Diagnostic Center, Heraklion, Greece; ^5^ Biology Center of the Czech Academy of Sciences, Budweis (Ceske Budejovice), Czechia

**Keywords:** machine learning, hematological malignancy, scoping review, artificial intelligence, radiology

## Abstract

**Background:**

Here, we conducted a scoping review to (i) establish which machine learning (ML) methods have been applied to hematological malignancy imaging; (ii) establish how ML is being applied to hematological cancer radiology; and (iii) identify addressable research gaps.

**Methods:**

The review was conducted according to the Preferred Reporting Items for Systematic Reviews and Meta-Analysis Extension for Scoping Reviews guidelines. The inclusion criteria were (i) pediatric and adult patients with suspected or confirmed hematological malignancy undergoing imaging (*population*); (ii) any study using ML techniques to derive models using radiological images to apply to the clinical management of these patients (*concept*); and (iii) original research articles conducted in any setting globally (*context*). Quality Assessment of Diagnostic Accuracy Studies 2 criteria were used to assess diagnostic and segmentation studies, while the Newcastle–Ottawa scale was used to assess the quality of observational studies.

**Results:**

Of 53 eligible studies, 33 applied diverse ML techniques to diagnose hematological malignancies or to differentiate them from other diseases, especially discriminating gliomas from primary central nervous system lymphomas (n=18); 11 applied ML to segmentation tasks, while 9 applied ML to prognostication or predicting therapeutic responses, especially for diffuse large B-cell lymphoma. All studies reported discrimination statistics, but no study calculated calibration statistics. Every diagnostic/segmentation study had a high risk of bias due to their case–control design; many studies failed to provide adequate details of the reference standard; and only a few studies used independent validation.

**Conclusion:**

To deliver validated ML-based models to radiologists managing hematological malignancies, future studies should (i) adhere to standardized, high-quality reporting guidelines such as the Checklist for Artificial Intelligence in Medical Imaging; (ii) validate models in independent cohorts; (ii) standardize volume segmentation methods for segmentation tasks; (iv) establish comprehensive prospective studies that include different tumor grades, comparisons with radiologists, optimal imaging modalities, sequences, and planes; (v) include side-by-side comparisons of different methods; and (vi) include low- and middle-income countries in multicentric studies to enhance generalizability and reduce inequity.

## 1 Introduction

Radiology is at the forefront of applied artificial intelligence (AI) due to the digitization and archiving of vast numbers of radiology images coupled with the availability of high-performance, low-cost computers. Machine learning (ML) is a branch of AI that uses computational resources to detect underlying patterns in high-dimensional, “big” data. ML is increasingly used in radiology (1) and other medical specialties requiring predictions such as predicting hypertension (2) or chronic kidney disease risk (3). ML algorithms have now been developed and tested in almost every radiological subspecialty, including X-ray interpretation to reduce turnaround times, analyzing screening mammograms for breast cancer, segmenting pulmonary embolism in CT angiography, and brain tumor segmentation with MRI (4). This is also true in hematology, where ML has been applied to hematological malignancy radiology and to cytology, molecular genetics, and cytogenetics (5). However, there is currently no comprehensive assessment of ML in hematological oncology radiology.

This scoping review focuses on the application of ML to the diagnosis and prediction of hematological malignancies using radiological approaches. It also serves as an exemplar of the challenges faced when applying ML to diagnostic and predictive problems in hematology and oncology where computational approaches would benefit the specialty. ML has already been applied to other areas of hematology such as cytomorphometry (identifying and characterizing cell populations based on their morphology after imaging), cytogenetics (identifying and selecting individual chromosomes and chromosomal abnormalities), and immunophenotyping (identification and characterization of flow cytometry cell populations based on light-scattering properties and antigen expression patterns) (5). ML has also been applied to every other area of clinical oncology including automated histopathological analysis, molecular subtyping, prognostication and predicting responses to therapies using clinical and/or biomarker data (genomic and transcriptomic), and precision oncology (such as the inference of genomic traits from histopathological images) (6). Therefore, many of the principles underlying the application of ML to hematological oncology radiology—and the challenges and conclusions—will be of interest to every clinician since all patient care is likely to be impacted by rapid developments in this field.

### 1.1 A brief overview of machine learning and some important definitions

For an excellent overview of ML in radiology, see (4). For the purposes of this scoping review, ML refers to the automatic detection, or *learning*, of patterns and associations within data using computational resources and *algorithms* (a series of steps, often mathematical equations, designed to solve a problem). In the context of imaging, by applying an ML algorithm to images (such as CT, MRI, or FDG-PET images) and given some prior knowledge about these images (such as whether they contain a benign or malignant tumor), the algorithm can *learn* from training images and apply this knowledge to unseen images to make a prediction. Hopefully, as the parameters in the equations are optimized, the prediction improves, i.e., the algorithm learns.

From this basic description of ML, we can describe a set of definitions to help understand the ML literature. The *model* represents the rules, numbers, and any other algorithm-specific data structures required to make predictions after running an ML algorithm on training data. *Labels* refer to the “correct” answer assigned to the images or parts of the images, such as the presence of a lymphoma or the boundary of a diseased node. When the label is assigned to a group of pixels, this is known as *classification*. *Training* refers to the ML model learning from labeled data until it can no longer improve, while the *validation set* refers to a second set of data to which the model is applied to provide an unbiased estimate of the skill of the final tuned model when comparing or selecting between final models. In some cases, a third set of examples is used for “real-world” testing, known as *testing*, although, confusingly, the terms “validation” and “testing” are often used interchangeably. The application of the model to a third (preferably independent) set of data helps to ensure that the model *generalizes* with high accuracy to new, unseen data. Since the label in many medical applications is known (i.e., presence or absence of lymphoma), these models tend to be *supervised* as opposed to *unsupervised*, where the output is unknown and generated by the model itself. Finally, the data used to construct ML models are called *features*, which might be pixel values or variations, edge strengths, or any other numerical value derived by *radiomics—*a method that extracts huge numbers of features from medical images using data-characterization algorithms. For medical applications, non-imaging features may also be used such as a laboratory test value or clinical parameter such as age or gender.

Many different types of the ML model have been used in radiology research. These include decision trees [DTs; and their adaptation, random forests (RFs)], support vector machines (SVMs), and artificial neural networks (ANNs), complete descriptions of which can be found in (4). For the purposes of this review, it is useful to know that SVMs tend to generalize well to unseen data and work well with complex (multidimensional) data but can be hard to interpret (7, 8) and that ANNs—inspired by neural connections in the human brain—are computationally expensive and represent a “black box.” That is, the way in which they performed the classification is not known; thus, they are therefore difficult to interpret clinically (7, 8).

### 1.2 The clinical imperative for using ML in the hematological cancer radiology

There are several intuitive areas in which ML can be applied to hematological malignancy imaging: diagnosis, segmentation, and prognostication or therapeutic response prediction.

#### 1.2.1 Clinical rationale for developing ML-based diagnostic models

While the morphological features of lymphomas and other benign and malignant neoplasms and inflammatory diseases are usually easy to distinguish, diagnostic difficulties can arise in certain cases when these appearances overlap. For example, most glioblastomas (GBMs) exhibit ring-like or heterogeneous enhancement on MRI with the areas of hypointense necrosis. Primary central nervous system lymphomas (PCNSLs) show uniform enhancement and low cerebral blood volumes (CBVs). Despite these diagnostic features, atypical cases can cause diagnostic difficulty (for example, non-necrotic GBMs or necrotic PCNSLs) (9, 10). Furthermore, “hypervascular PCNSLs” have high CBVs that mimic GBM (11). While biopsy can resolve the diagnostic dilemma, this is invasive, can cause complications, and may be non-diagnostic, particularly if steroids have lysed lymphoma cells (12). While complex imaging protocols including quantitative diffusion-, perfusion-, and susceptibility-weighted imaging; texture analysis; or their combination may increase diagnostic yield, a sufficiently accurate automated analysis of routine diagnostic images with ML could help diagnostic decision-making. It is essential to make the correct diagnosis, with minimal resources, and with minimal harm to the patient, since PCNSL and GBM are managed differently: methotrexate-based chemotherapy with or without radiotherapy for the former and surgical resection with radiochemotherapy for the latter (10, 13). Similar diagnostic difficulties occur at other sites including differentiating thymic neoplasms (usually surgical treatment) from lymphoma (medical management) (14) and differentiating breast carcinoma from lymphoma (15).

A second major application of ML techniques is to improve the detection and monitoring of hematological malignancies for accurate diagnosis, treatment, and staging. For example, the FDG-PET/CT images of lymphomas and multiple myeloma can be difficult to interpret due to low avidity, unusual distribution patterns [particularly diffuse disease in multiple myeloma (16) and leukemia (17)], or motion/attenuation artifacts, especially for inexperienced readers. Algorithms to support diagnostic decision-making would therefore be helpful (16, 18).

#### 1.2.2 Clinical rationale for developing ML-based segmentation models

Total metabolic tumor volume (TMTV)—the quantification of the metabolically active volume of tumor assessed by FDG-PET/CT—is prognostic for many hematological malignancies including Hodgkin (19) and non-Hodgkin (20) lymphomas. Some patients are resistant to therapy or relapse, and it is difficult to identify this subset with existing prognostic indices [such as the international prognostic index (IPI) or international prognostic score (IPS) for HL]. New, accurate prognostic indices to drive personalized treatment approaches are still needed. While TMTV might form a valuable component of a prognostic algorithm, its computation currently requires the marking, often manually, of many regions of interest (ROIs). This is time-consuming and operator dependent, also contributing to error, and several non-standardized approaches are currently used to threshold and segment lesions, e.g., SUV ≥41%, SUV ≥2.5, or SUV ≥ mean liver uptake. ML approaches lend themselves to automating and standardizing this task by learning the most important imaging features while subtracting physiological uptake. This is challenging since the algorithms must handle low-resolution PET and the partial volume effect (loss of signal in small areas due to limited resolution), the high distribution variability of lesions, and the recognition and subtraction of physiological uptake in different organs.

#### 1.2.3 Clinical rationale for developing ML-based prognostic/predictive models

As noted above, not all patients respond to therapy; for example, only ~60% of diffuse large B-cell lymphoma (DLBCL) patients benefit from current therapies and ~15% experience primary treatment failure and a median survival of under 1 year (21). Identifying these patients would allow the tailored addition of emerging therapies such as chimeric antigen receptor T-cell (CAR-T) therapy in patients most likely to fail first-line therapy. However, it is becoming increasingly clear that identifying and quantifying variability in and between lesions in the same patient, i.e., tumor heterogeneity, is as important as quantifying the *amount* of tumor since the molecular and microenvironmental differences reflected by this heterogeneity contribute to progression (i.e., prognosis) and therapeutic responses (22). Since ML can detect underlying patterns in high-dimensional data that are invisible to humans, it is hypothesized that ML can better interpret the quantitative and spatial data embedded in radiology images reflecting tumor heterogeneity. Associating these previously unseen patterns with the outcomes of interest such as survival or therapeutic response is then expected to identify the future clinical course of individual patients.

## 2 Objectives

Radiologists managing patients with hematological cancers are faced with clinical problems defined by known and unknown imaging features corresponding to disease states or clinical outcomes. This lends itself to the application of supervised ML techniques to develop diagnostic or predictive models. We therefore performed a scoping review of studies using ML techniques on radiology images to (i) establish which, if any, ML methods have been applied to hematological malignancy imaging; (ii) establish the main applications of ML in hematological cancer radiology; and (iii) identify research gaps that must be addressed to advance the field.

## 3 Methods

The Preferred Reporting Items for Systematic Reviews and Meta-Analysis Extension for Scoping Reviews (PRISMA-ScR) guidelines were applied (see **Supplementary File 1** for the checklist) (23).

### 3.1 Rationale for performing a scoping review

A scoping review methodology (24) was chosen to map the available evidence since initial literature assessment showed that (i) evidence on ML in hematological cancer radiology is only just emerging; thus, a first impression of this field of research was appropriate and (ii) available studies are highly heterogenous and use many ML methods for several purposes/applications (24). The scoping approach therefore allowed us to (i) identify the available evidence; (ii) clarify key concepts and definitions; (iii) examine how research is being conducted, in which populations, and for what purposes; and (iv) identify knowledge gaps (24).

### 3.2 Inclusion and exclusion criteria

Using the population, concept, and context approach (25), inclusion criteria were (i) pediatric and adult patients with a suspected or confirmed hematological malignancy undergoing imaging (*population*); (ii) any study using ML techniques to derive models using radiological images for clinical benefit (*concept*); and (iii) original research articles conducted in any setting globally (*context*). All modeling approaches defined as ML in the respective papers (such as logistic regression) were included, with the assumption that the data analysis was almost wholly computer driven.

Exclusion criteria were any study in which an ML model was not defined and outcomes were not defined (for prognostic/predictive studies) and/or not written in English.

### 3.3 Literature search

The PubMed database was searched to identify literature meeting the study criteria published in English from inception to 1 October 2021. The search term used was [((machine learning) OR (artificial intelligence) OR (decision tree) OR (neural network) OR (random forest) OR (support vector machine) OR (radiomics)) AND ((radiology) OR (imaging) OR (tomography) OR (magnetic resonance)) AND ((hematological malignancy) OR (lymphoma) OR (myeloma) OR (leukemia))].

Articles were included if they used supervised ML techniques to interpret diagnostic radiology images in any patient with a hematological malignancy. Commentaries, editorials, letters, or case reports were excluded. All abstracts identified by the initial search were screened for inclusion and checked for accuracy. Disagreements over inclusion were resolved by consensus between the researchers.

Data were extracted from papers meeting the inclusion criteria to populate tables prior to analysis. The data of interest included study population characteristics; imaging modalities; ML algorithms used; the methods of model validation; performance measures such as accuracy, sensitivity, specificity, and AUC; and direct comparison to other algorithms or radiologist performance.

### 3.4 Quality assessment

No current quality assessment tool specifically addresses ML methodology, although the Checklist for Artificial Intelligence in Medical Imaging (CLAIM) is a recently published guideline that helps authors applying ML to medical imaging applications present their research optimally (26).

Therefore, for diagnostic studies (including segmentation analyses), the following CLAIM items were used to assess each domain of the Quality Assessment of Diagnostic Accuracy Studies 2 (QUADAS-2) criteria (27): (1) data sources, the selection of data subsets and how missing data were handled in the patient selection risk of bias; (2) the measures of significance and uncertainty and robustness or sensitivity analysis in the index test risk of bias; (3) sufficient detail to allow replication about the definition of ground truth, rationale for choosing the reference standard, qualifications, and preparation of annotators for the source of ground truth annotations in the reference standard test risk of bias; and (4) validation or testing on external data when assessing concerns regarding the applicability of the index test.

The Newcastle–Ottawa Scale (NOS) (28) was used to assess the quality of prognostic/predictive studies with scores converted to AHRQ standards, i.e., good quality: three or four stars in the selection domain AND one or two stars in the comparability domain AND two or three stars in the outcome/exposure domain; fair quality: two stars in the selection domain AND one or two stars in the comparability domain AND two or three stars in the outcome/exposure domain; and poor quality: zero or one star in the selection domain OR zero star in the comparability domain OR zero or one star in the outcome/exposure domain.

## 4 Results and discussion

### 4.1 Main findings

#### 4.1.1 Identified risk models

**Figure 1:**
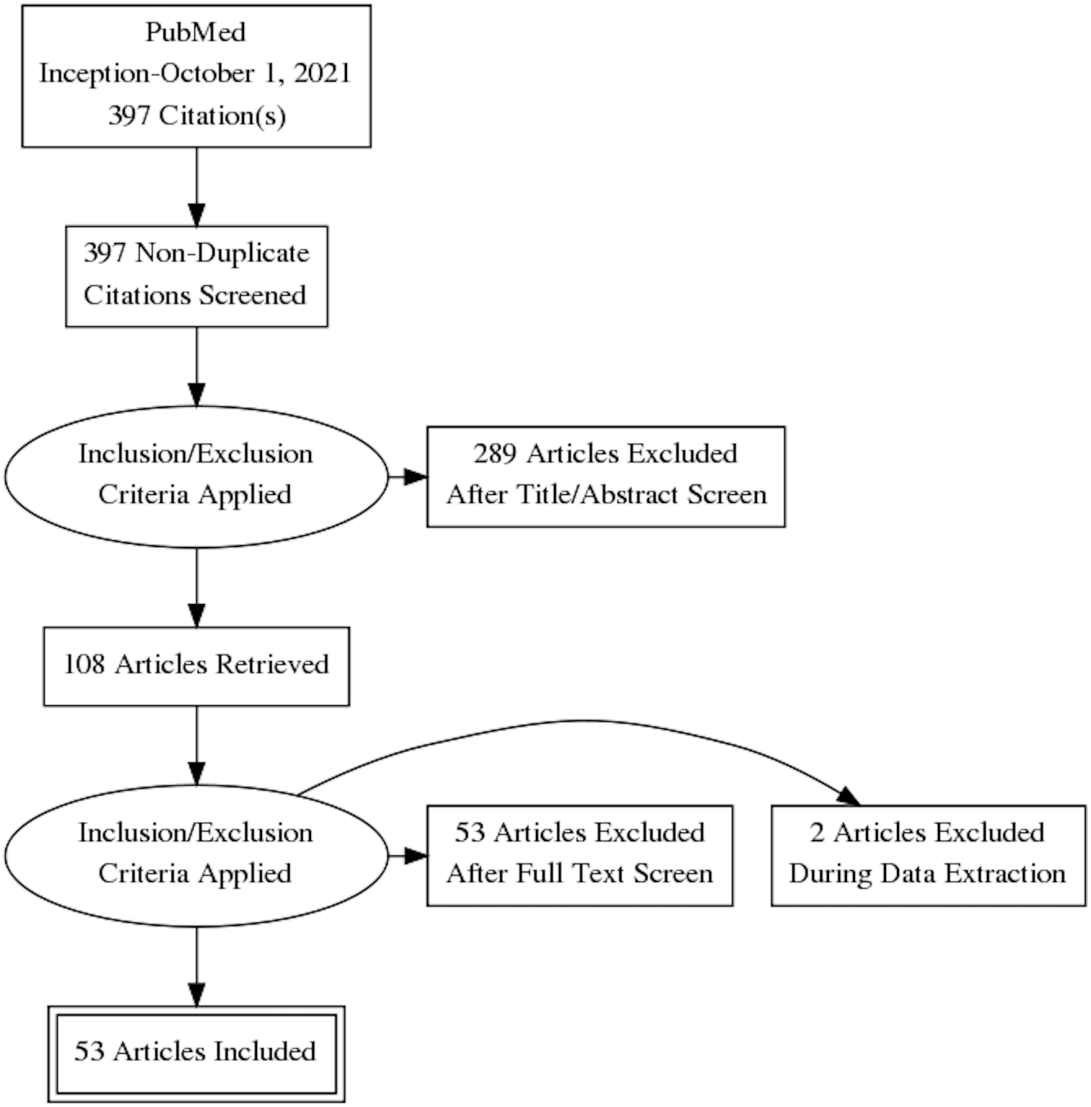
The Preferred Reporting Items for Systematic Revsiews and Meta-Analysis (PRISMA) flowchart depicting the search strategy. Figure prepared using the PRISMA Flow Diagram Generator (http://prisma.thetacollaborative.ca).

A total of 397 studies were identified, of which 53 studies met the inclusion criteria (**Table 1** **;** **Figure 1**). The most common reasons for exclusion were (i) ML was not the primary analytical methodology or the analysis was not computer driven and (ii) the studies used features from non-imaging data such as histopathological or cytology images.

**Table 1:** Studies applying machine learning to the diagnosis of lymphoma or to distinguish it from another malignancy.

Reference	Country	Patient populations	Imaging modality	Learning algorithm	Features	Diagnostic aim	Results	Validation	Comparison with radiologist (or other) assessment
Alcaide-Leon et al., 2017 (29)	Canada	Glioma n=71 PCNSL n=35	PET	SVM classifier	11 first-order and 142 second-order texture features	Distinguish enhancing glioma from PCNSL	SVM: AUC 0.877 (0.798-0.955)	No	Reader 1: AUC 0.878 (0.807-0.949) Reader 2: AUC 0.899 (0.833-0.966) Reader 3: 0.0.845 (0.757-0.933) SVM significantly non-inferior to human interpretation
Chen et al., 2020 (30)	China	GBM n=76 PCNSL n=62	T1 contrast-enhanced MRI	LDA, SVM, and LR classifiers	Automatic feature extraction using lifeX; different features according to: -Distance correlation - RF - LASSO - eXtreme gradient boosting (Xgboost) - Gradient boosting decision tree (GBDT)	Establish and train the models to discriminate GBM from PCNSL with radiomics features extracted from T1C imaging	For LDA-based models, the AUCs in the validation group were 0.978, 0.964, 0.977, 0.750, and 0.956; for the SVM-based models, the AUCs were 0.959 and 0.822; and for LR-based models, the AUCs were 0.933 and 0.975	Cohort randomly split 4:1	N/A
Chen et al., 2018 (31)	China	GBM=66 PCNSL=30	T1 contrast-enhanced MRI	CNN for segmentation, SIFT for feature extraction, GA to extract SIFT features, and SVM	496 after GA	Establish and train the models to discriminate GBM from PCNSL with radiomics features extracted from T1C imaging	AUC 99.1 cross-validation cohort, AUC 98.2 independent cohort	Cohort randomly split 2:1	N/A
Ferjaoui et al., 2021 (32)	Tunisia	n=50	MRI	End-to-end automated segmentation, feature extraction, and classification (back-propagation ANN, SVM, K-NN, relevance vectors machine, and the RF compared to deep learning based on a CNN).	72 extracted features	Detect evolving lymphoma and residual disease	Accuracy 97%	80:20 random split	N/A
Hou et al., 2021 (33)	China	Idiopathic orbital inflammation n=28 ocular adnexal lymphoma n=28	Contrast-enhanced MRI	SVM with linear kernel	160 textural features encoded into BOF representation	Establish and train a model to discriminate IOI from OAL	AUC 0.803 (0.725-0.880) with a sensitivity 71.4%, specificity 90.5%	5-fold cross-validation and random 4:1 split	Inexperienced radiologist: sensitivity of 60.7%, specificity 57.1% Experienced radiologist: sensitivity of 75.0%, specificity 67.9%
Kang et al., 2018 (34)	South Korea	Training n=70 glioblastomas n=42 PCNSL Test n=28 GBM, n=14 PCNSL	MRI	K-NN, naiüve Bayes classifier, decision tree, LDA, RF, adaptive boosting, linear SVM, and radial basis function support vector machine classifiers	Selected from 17 first-order features, 7 volume and shape features, 162 texture features, and 1,432 wavelet features	Establish and train the models to discriminate GBM from PCNSL with radiomics features extracted from routine MRI	Training set AUCs 0.910–0.983 depending on classification and feature selection method Test set AUC 0.787-0.946 depending on classification and feature selection method	External validation set from another center Used heterogenous MRI protocol, confirming robustness	Human readers AUC 0.825–0.930
Kim et al., 2018 (35)	South Korea	Training n=86 GBM, n=37 PCNSL Testing n=57 GBM, n=28 PCNSL	MRI	Minimum redundancy maximum relevance (mRMR) algorithm and LASSO for feature selection, logistic classifier, SVM, and RF for classification	15 selected from 127 radiomics features	Establish and train the models to discriminate GBM from PCNSL with radiomics features extracted from routine MRI	LR AUC 0.979 discovery, 0.956 independent validation SVM AUC 0.997 discovery, 0.947 independent validation RF AUC 1.00 discovery, 0.953 independent validation	Validation cohort PCNSL had atypical appearances	N/A
Kirienko et al., 2020 (14)	Italy	n=55 thymic neoplasm n=53 lymphoma	CT	LDA	Selected from 41 LIFEx radiomic features	Establish and train the models to discriminate thymic neoplasms from thymic lymphoma with radiomics features	AUC 0.90–0.97 training, 0.70–0.95 validation, depending on final model AUC clinical model 0.95	Split sample	N/A
Kunimatsu et al., 2018 (36)	Japan	Training n=44 GBM n=15 PCNSL Testing n=11 GBM n=5 PCNSL	T1 contrast-enhanced MRI	SVM classifier	67 texture features	Establish and train the models to discriminate GBM from PCNSL with features extracted from routine MRI	Training: AUC 0.99 (0.86-1.00) training with Gaussian kernel, AUC 0.87 (0.77-0.96) training with linear kernel 75% accuracy on testing	Sample split by time periods, therefore potentially biased	N/A
Li et al., 2019 (17)	China	n=41 (35 training, 6 testing) with acute leukemia	FDG-PET/CT	Random forest	2 PET and 1 CT feature	Identify bone marrow involvement in patients with suspected relapse of leukemia	ML: 87.5%, 89.5%, and 88.6% sensitivity, specificity, and accuracy *vs*. 62.5%, 73.7%, and 68.6% for visual inspection 83.3% accuracy independent validation	Small validation cohort split by time period (same institution), therefore potentially biased	N/A
Liu et al., 2012 (37)	UK	n=10 GBM, n=8 PCNSL	T1 contrast-enhanced MRI	Hybrid method with wavelet analysis, Gabor wavelet analysis, SVM, and LDA	2 features	Establish and train the models to discriminate GBM from PCNSL with features extracted from routine MRI	98.9% accuracy	Cross-validation	88.9% from radiology reports
Martínez-Martínez et al., 2016 (38)	Czech Republic	n=127 MM	CT	K-NN and SVM classifier	2 features	Differentiating patients with and without bone marrow infiltration	AUC 0.996 ± 0.009 with SVM	4:1 split	Compares favorably with 0.867 for blood serum testing
Mayerhoefer et al., 2020 (39)	USA	n=97 mantle cell lymphoma patients	FDG-PET/CT	PCA for feature selection Multilayer perceptron NN for classification	5 radiomic components	Differentiating patients with and without bone marrow involvement	AUC up to 0.81 Laboratory parameters improved performance	Split into training (n=68) and test (n=29) cohorts	N/A
McAvoy 2021 (40)	USA	GBM (n = 160) and PCNSL (n = 160)	MRI	3 CNN models	Automated feature selection	Establish and train the models to discriminate GBM from PCNSL with features extracted from routine MRI	AUCs 0.92-0.95	Images split into training (n=189) and test (n=59) sets	N/A
Mesguich et al., 2021 (16)	France	n=30 multiple myeloma patients	FDG-PET/CT	RF	5 radiomic features	Identifying diffuse disease	AUC 0.90, accuracy 91%, 80% accuracy in “independent” test set	Independent test set (n=10 from original n=30)	Not fully independent validation, even though stated as such
Nakagawa et al., 2018 (41)	Japan	n=45 GBM, n=25 PCNSL	Contrast-enhanced MRI	Univariate logistic regression and multivariate eXtreme gradient boosting-XGBoost	48 features	Establish and train the models to discriminate GBM from PCNSL with features extracted from routine MRI	AUC 0.98	10-fold cross-validation	0.84 and 0.79 for radiologists
Ou et al., 2019 (15)	China	n=25 breast cancer n=19 breast lymphoma	FDG-PET/CT	LDA	Up to 15 clinical, SUV, and radiomic features	Establish and train the models to discriminate primary breast carcinoma from breast lymphoma	AUC 0.87 for best performing model	4:1 split with 10-fold cross-validation	N/A
Priya et al., 2021 (42)	USA	n=97 GBM, n=46 PCNSL	MRI	First-order histogram features and multiple linear and non-linear ML classifiers	Multiple linear and non-linear ML classifiers	Establish and train the models to discriminate GBM from PCNSL with features extracted from routine MRI	AUCs between 0.74 and 0.92	5-fold cross-validation	N/A
Seidler et al., 2019 (43)	Canada	n=10 patients er group (number of nodes shown in brackets) HNSCC with metastases (n = 31) HNSCC with benign nodes (n = 145) lymphoma (n = 65) inflammatory (n = 29) no lymph node history (n = 142)	Dual-energy CT	RF and gradient boosting	6 texture features	Prediction accuracy for distinction of lymphoma from not only normal nodes but also inflammatory from normal and benign *vs*. malignant	AUC 0.95 both training and testing (RF) and 0.99 and 0.96 for gradient boosting	7:3 split	N/A
Sibille et al., 2019 (18)	Germany and USA	n=327 patients with lymphoma	FDG-PET/CT	CNN	Combinations of CT, PET, and maximum intensity projection features	Localization and diagnosis	AUC 0.97, 0.84, and 0.88 for lymphoma localization to body part, organ, and subregion, respectively 0.95 classification accuracy	Random split 20:60:20 validation:training:testing	N/A
Suh et al., 2018 (44)	South Korea	n=54 PCNSL n=23 GBM	MRI	Recursive feature elimination and random forest (RF) analysis	80 radiomics features	Establish and train the models to discriminate GBM from PCNSL with features extracted from routine MRI	Mean AUC of ML 0.921	Nested cross-validation	0.70-0.76 for three human readers
Swinburne et al., 2019 (45)	USA	n=9 GBM n=9 metastasis n=8 PCNSL	MRI	Support vector classifier (SVC) and multilayer perceptron (MLP) models	Automated feature selection	Establish and train the models to discriminate GBM, PCNSL, and metastases with features extracted from routine MRI	Maximum 69.2% accuracy for ML	Unlabeled cases using the leave-one-subject-out method	65.4% and 80.8% for two human readers 19% increase in diagnostic yield when added to routine human interpretation
Tomita et al., 2021 (46)	Japan	n=17 nasopharyngeal cancers and n=17 nasopharyngeal lymphomas	CT	SVM	5 combined texture features	Differentiate between nasopharyngeal cancer and nasopharyngeal malignant lymphoma	AUC 0.80	50 repetitions of 5-fold cross-validation	N/A
Wang et al., 2021 (47)	China	n=186 MM patients	CT	U-net for segmentation, Faster R-CNN for detection	U-net for segmentation	Segmentation and lesion labeling	0.96 classification accuracy	None	N/A
Wang et al., 2021 (48)	China	n=154 (n=74 SCC and n=80 lymphomas)	MRI	SVM	5 radiomics features	Discriminate sinonasal primary lymphoma and SCC	AUC of 0.92	80:20 random split	AUCs of radiologists 0.76–0.80
Xia et al., 2021 (49)	China	n=289 PCNSL and n=153 GBM	MRI	CNN	Single parameter (CE-T1WI, FLAIR, and ADC) and multiparameter (image- and decision-level fusion) models	Establish and train the models to discriminate GBM from PCNSL with features extracted from routine MRI	Accuracies between 0.7 and 0.90	5-fold cross-validation	Not significantly different to experienced radiologist and radiomics model
Xiao et al., 2018 (50)	China	n=60 GBM n=22 PCNSL	MRI	SVM, NB, RF	3 features	Establish and train the models to discriminate GBM from PCNSL with features extracted from routine MRI	AUC 0.85 LR AUC 0.90 NB AUC 0.87 SVM	10-fold cross-validation	N/A
Xiong et al., 2021 (51)	China	n=47 MM, n=60 metastases	MRI	SVM, RF, NB, K-NN, and ANN	13 features in T1WI images and 9 features in T2WI images	Differentiating between multiple myeloma and metastasis subtypes in lumbar vertebrae	ANN best performance (AUC 0.61) Less accurate at predicting metastasis subtypes	10-fold cross-validation, 70:30 split	N/A
Xu et al., 2018 (52)	Germany	n=12 MM patients	FDG-PET/CT	CNN	Automated feature selection	Bone lesion detection	Sensitivity 73.5%, Specificity 99.5%	3-fold cross-validation	N/A
Yamasaki et al., 2013 (53)	Japan	n=20 GBM n=20 PCNSL	MRI	SVM	Luminance range thresholding	Establish and train the models to discriminate GBM from PCNSL with features extracted from routine MRI	91.1% accuracy	Leave-one-out cross-validation	N/A
Yang et al., 2017 (54)	China	n=58 GBM n=37 PCNDL	MRI	SVM	Manual feature extraction	Establish and train the models to discriminate GBM from PCNSL with features extracted from routine MRI	96.4% accuracy	Leave-one-out cross-validation	N/A
Yun et al., 2019 (55)	South Korea	n=73 GBM, n=50 PCNSL training; n=18 GBM, n=12 PCNSL internal validation; n=28 GBM, n=14 PCNSL external validation	MRI	SVM, GLM, or RF or multilayer perceptron (MLP) network End-to-end CNN	Manual feature extraction	Establish and train the models to discriminate GBM from PCNSL with features extracted from routine MRI	AUC GLM 0.94 AUC MLP 0.99 AUC CNN 0.73 in training; MLP AUC 0.95 in external validation but CNN only 0.49	Internal and external validation	N/A
Zhang et al., 2021 (56)	China	Solitary and multiple cerebral GBM (n = 97), PCNSL (n = 92), and tumefactive demyelinating lesion (n = 72)	MRI	End-to-end deep learning model	Automated feature selection	Differentiate solitary and multiple cerebral GBM, PCNSL, and tumefactive demyelinating lesion	AUCs of GBM, PCNSL, and tumefactive demyelinating lesion were 1.00 (95% confidence interval [CI]: 1.000–1.000), 0.96 (95% CI: 0.923–1.000), and 0.954 (95% CI: 0.904–1.000), respectively	None	N/A

ANN, artificial neural network; AUC, area under the (receiver operating characteristics) curve; BOF, bag of features; CNN, convolutional neural network; CT, computed tomography; FDG, fluorodeoxyglucose; GA, genetic algorithm; GBM, glioblastoma multiforme; GLM, generalized linear model; HNSCC, head and neck squamous cell carcinoma; K-NN, K-nearest neighbors; LDA, linear discriminant analysis; LASSO, least absolute shrinkage and selection operator; LR, logistic regression; ML, machine learning; MRI, magnetic resonance imaging; N/A, not applicable; NB, naïve Bayes; NN, neural network; PCNSL, primary central nervous system lymphoma; PET, positron emission tomography; R-CNN, region-based convolutional neural network; RF, random forest; SCC, squamous cell carcinoma; SIFT, scale-invariant Fourier transform; SUV, standardized uptake value; SVM, support vector machine.

All studies were retrospective studies conducted between 2012 and 2021, and 39 (74%) were published in the last 3 years. There were 14 studies conducted in China, 15 in the UK/Europe, 11 in North America, 5 in South Korea, 4 in Japan, and 2 in the rest of the world (Tunisia and Turkey); only two were multicenter, multinational (Germany/USA and Denmark/USA) studies.

There were 33 studies that applied ML techniques to diagnose a hematological malignancy or to differentiate it from another disease state or malignancy (**Table 1**); 11 applied ML for segmentation tasks alone (**Table 2**), while 9 applied ML to the problem of prognostication or predicting responses to therapy (**Table 3**). These subgroups are considered separately below.

**Table 2:** Studies applying machine learning to segmentation tasks.

Reference	Country	Number of patients	Validation	Disease	Imaging modality	Learning algorithm	Ground truth	Results	Notes
Blanc-Durand et al., 2020 (57)	France	n=733, n=639 for training and n=94 for validation	n=94 for validation	DLBCL	FDG-PET/CT	3D deep CNN based on open-source Python libraries	Ground truth masks were manually obtained after a 41% SUV_max_ adaptive thresholding of lymphoma lesions	Mean DSC and Jaccard coefficients (± standard deviation) in the validations set were 0.73 ± 0.20 and 0.68 ± 0.21, respectively R^2^ of 0.88 and 0.82 in first and second cohorts TMTV was underestimated in both first and second validation cohorts SUV_max_ currently predicted in 83% of cohort 1 and 33% cohort 2 compared with 92% for two human readers	
Copobianco et al., 2020 (58)	France	n=280	Nil	DLBCL	FDG-PET/CT	CNN trained on independent cohort	The reference TMTV was measured by two experienced readers using independent semiautomatic software after 41% SUV_max_ adaptive thresholding of lymphoma lesions	Algorithm significantly correlated with reference TMTV (rho=0.76, p<0.001) Dice score 0.73 Predictive of PFS and OS (PFS hazard ratio: 2.4 and 2.6 for AI-based and reference TMTVs; OS hazard ratio: 2.8 and 3.7 for AI-based and reference TMTVs, respectively)	Independent validation of CNN presented in (18)
Grossiord et al., 2017 (59)	France	n=43	Leave-one-out cross-validation	Lymphoma, unspecified	FDG-PET/CT	Random forest based on intensity, shape, textural, and spatial features	41% SUV_max_ in manually placed ROIs	92% detection of lesions, retrieved 75% of tumor volume, but overestimated tumor volume by 35%	
Hu et al., 2020 (60)	Germany	n=83	80:20 split	NK/T-cell lymphoma	FDG-PET/CT	Coarse-to-fine adversarial segmentation network	Manual by two radiologists, otherwise undefined	Dice coefficient 0.7115 ± 0.132	
Jemaa et al, 2020 (61)	USA	n=2266 DLBCL training n=1124 FL testing	n=1124 FL testing	NHL and NSCLC	FDG-PET/CT	Cascaded 2D to 3D CNN	Manual by radiologists, otherwise undefined	Dice score 0.895 training, 0.886 testing 0.97 and 0.96 TMTV and SUV_max_ correlations with radiologist ground truth	
Pennig et al., 2021 (62)	Germany	n=43 patients with PCNSL	None	PCNSL	MRI	3D CNN	Manual segmentation, otherwise undefined	High volumetric correlation between automated and manual segmentations was observed (TTV: r = 0.88, P < 0.0001; core: r = 0.86, P < 0.0001) Median DSC: 0.76	
Sadik et al., 2018 (63)	Sweden	n=80 lymphoma patients (testing)	n=6 validation	Lymphoma	FDG-PET/CT	CNN	Manual segmentation, otherwise undefined	Dice scores 0.95 for both algorithm and radiologists for liver segmentation	
Weisman et al., 2020 (64)	USA, Denmark	n=63 newly diagnosed NHL n=27 newly diagnosed DLBCL	5-fold cross-validation	NHL and DLBCL	FDG-PET/CT	Thresholding Clustering Adaptive region growing DeepMedic U-net	Manual segmentation, otherwise undefined	DeepMedic, achieved the highest performance across all evaluation metrics and was comparable to physician assessment (DSC approximately 0.70)	Multiple methods, including a 3D CNN, clustering, and an iterative threshold method, achieved both good lesion-level segmentation and patient-level quantification performance. However, thresholding outside limits defined by inter-physician agreement
Weisman et al., 2020 (65)	USA	n=100 pediatric HL	5-fold cross-validation	HL	FDG-PET/CT	CNN (DeepMedic)	40% SUV_max_ in manually placed ROIs	Median Dice similarity coefficient between automated and physician contours was 0.86 Pearson’s *R*=0.95 between physician and ML SUV_max_. Pearson’s *R*=0.88 between physician and ML MTV, although slightly underestimated	Excellent agreement with reference physician PET segmentation
Yu et al., 2018 (66)	France	n=8 lymphoma patients	Not stated	Lymphoma (unspecified)	FDG-PET/CT	Conditional random fields	41% SUV_max_ in manually placed ROIs	100% detection, Dice indices 84.4% compared to manual segmentation	
Yuan et al., 2021 (67)	China	n=45	15-fold cross-validation	DLBCL	FDG-PET/CT	CNN	Not stated	Mean Dice similarity coefficient (DSC) of 73%	

3D, three dimensional; CNN, convolutional neural network; CT, computed tomography; DLBCL, diffuse large B-cell lymphoma; DSC, Dice similarity coefficient; FDG, fluorodeoxyglucose; HL, Hodgkin lymphoma; MRI, magnetic resonance imaging; HNL, non-Hodgkin lymphoma; NSCLC, non-small cell lung cancer; PCNSL, primary central nervous system lymphoma; PET, positron emission tomography; ROI, region of interest; SUV, standardized uptake value; TMVM, total metabolic tumor volume.

**Table 3:** Studies applying machine learning for the prognosis/prediction of responses to therapy.

Reference	Country	Number of patients	Imaging modality	Learning algorithm	Features	Disease/study aim	Therapy	Outcome	Results	Validation	NOS (quality)
Coskun et al., 2021 (68)	Turkey	n=45	FDG-PET	Recursive feature elimination for feature selection and LR classifier	14 features	Predicting response to therapy in DLBCL	R-CHOP	Response	AUC 0.81	3-fold cross-validation	6 (fair)
Guo et al., 2021 (69)	China	n=167	FDG-PET	Weakly supervised deep learning	128 features automatically extracted	Prognosis in extranodal natural killer/T-cell lymphoma, nasal type	Methotrexate, etoposide, dexamethasone, and pegaspargase	PFS	AUC of 0.99 (training) and 0.88 (test)	n=64 training, n=20 test	8 (good)
Jamet et al., 2020 (70)	France	n=139	FDG-PET/CT	RSF	17 image features, 5 clinicopathological features	Multiple myeloma/evaluate prognostic value of baseline PET features	Eligible for transplant (randomized)	Survival	Final model used three features (treatment arm, hemoglobin, and SUV_max_ Bone Marrow) defined good and poor prognosis groups mean HR 4.3 ± 1.5.	Train-test sets and nested cross-validation	7 (poor)*
Jullien 2021 (71)	France	n=656	CT	CNN	N/A	Prognostication based on muscle hypodensity DLBCL	Obinutuzumab *vs*. rituximab (R) in combination with CHOP (cyclophosphamide, doxorubicin, vincristine, and prednisone) or ACBVP *(as above* with bleomycin and vindesine replacing vincristine) chemotherapy in newly diagnosed untreated DLBCL	OS, PFS	Dice coefficient of 0.97 ± 0.03 for segmentation Muscle hypodensity was associated with lower OS and PFS, respectively (HR = 2.80 (95% CI 1.58–4.95), *p* < 0.001, and HR = 2.22 (95% CI 1.43–3.45), *p* < 0.001)	n=190 for training, n=670 for validation	9 (good)
Liu et al., 2021 (72)	China	n=37 HRC patients and n=52 non-HRC patients	MRI	Support vector machine, random forest, logistic regression (LR), decision tree, *k*-nearest neighbor, and XGBoost	Top three features in T1, T1, and T1/T2 images selected from 217 after three feature selection steps	Detecting HRC patients with MM	N/A	HRC status	LR two-sequence model outperformed other models (AUC 0.84)	10 repetitions of 5-fold cross-validation	8 (good)
Mayerhoefer 2019 (73)	USA	n=107 mantle cell lymphoma	FDG-PET/CT	Multilayer perceptron neural network in combination with logistic regression analyses for feature selection	SUV_mean_, entropy, lactate dehydrogenase level (LDH), white blood count (WBC), Ki-67 index and ECOG performance status	Prognostication	Scheduled to receive anti-CD20 immunotherapy	Progression-free survival	AUC 0.83 for predicting 2-year survival *vs*. 0.73 for radiomic features alone	7:3 random split	9 (good)
Milgrom et al., 2019 (74)	USA	n=251 HL	FDG-PET/CT	SVM	5 radiomic features	Primary clinical outcome was refractory or relapsed HL	Chemo(radio)therapy	Refractory or relapsed HL	AUC 0.95 for model *vs*. 0.78 for MTV and TLG and 0.65 for SUV_max_	10:2 split	7 (good)
Morvan et al., 2019 (75)	France	n=66 MM from a multicenter study	FDG-PET/CT	Random survival forest (RFS) and variable importance (VIMP) for both feature selection and prediction	14 clinical, 6 conventional, and 110 textural features	Progression-free survival of MM patients	Lenalidomide, bortezomib, and dexamethasone (RVD) with or without autologous stem-cell transplantation, followed by lenalidomide maintenance	PFS	Average prediction error 0.36 compared to 0.43–61 for conventional approaches	10-fold cross-validation	9 (good)
Santiago et al., 2021 (76)	Canada	n=26 refractory patients *vs*. n=26 non-refractory DLBCL patients	CT	RF	1,218 handcrafted radiomics features reduced to 66 + two additional features of nodal site and subjective necrosis	Predicting primary treatment failure (to R-CHOP) in DLBCL	R-CHOP	Primary treatment failure	AUC 0.83 and 0.79 for the two readers, respectively, compared to 0.56 and 0.52 for “subjective necrosis” alone	Two readers	6 (fair)

AUC, area under the (receiver operating characteristics) curve; CI, confidence interval; CNN, convolutional neural network; CT, computed tomography; DLBCL, diffuse large B-cell lymphoma; FDG, fluorodeoxyglucose; HRC, high-risk cytogenic; LR, logistic regression; MM, multiple myeloma; MRI, magnetic resonance imaging; NOS, Newcastle–Ottawa scale; OS, overall survival; PET, positron emission tomography; PFS, progression-free survival; R-CHOP, rituximab, cyclophosphamide, hydroxydaunorubicin hydrochloride (doxorubicin hydrochloride), vincristine (Oncovin) and prednisone; RF, random forest; RSF, random survival forest; SUV, standardized uptake value; SVM, support vector machine.

* Zero stars in the comparability domain.

### 4.2 ML models for diagnostic purposes

#### 4.2.1 Applications

Of the 33 studies that applied ML techniques to diagnose a hematological malignancy or to differentiate it from another disease state or malignancy (**Table 1**), 18 were designed to establish and train ML models to discriminate gliomas [predominantly GBM from PCNSL (29–31, 34–37, 40–42, 44, 45, 49, 50, 53–56)] using features extracted from FDG-PET [one study (29),] or MRI (30, 31, 34–37, 40–42, 44, 45, 49, 50, 53–56) images. The remaining studies belonged to two major categories: those developing models to discriminate solid hematological malignancies from other benign and malignant lesions at other sites [nasopharyngeal carcinomas from nasopharyngeal lymphoma (46, 48), idiopathic orbital inflammation from ocular adnexal lymphoma (33), thymic neoplasm from thymic lymphoma (14), breast carcinoma from breast lymphoma (15), lymphoma from normal nodes (43), or multiple myeloma from bone metastases (51)] and those that detect the location of hematological malignancies either at diagnosis or during the disease course [location of (18) or evolving/residual lymphoma (32) or leukemia (17) or bone marrow involvement with multiple myeloma (16, 38, 47, 52) or mantle cell lymphoma (39)].

#### 4.2.2 Model development

Many ML techniques were used, and, in some studies, different modeling approaches were compared on the same dataset (30, 32, 34, 35, 42, 43, 50, 51, 55). Others developed models using a single approach or a combination of approaches in an end-to-end manner (31, 32, 55, 56). The following diverse ML approaches were used to discriminate lymphomas from other benign or malignant lesions: support vector machines (SVMs (29–31, 33–37, 46, 48, 50, 51, 53–55);), linear discriminant analysis (LDA (14, 15, 30, 34, 37);), logistic regression (LR (30);), artificial/convolutional neural networks (A/CNNs (31, 40, 45, 49, 51, 55, 56);), *k*-nearest neighbors (K-NNs (34, 51);), naïve Bayes classification (NB (34, 50, 51);), decision trees (DTs (34);), random forests (RFs (34, 35, 43, 44, 50, 51, 55);), adaptive boosting (34), and gradient boosting (41, 43). The ML approaches used to detect the location of hematological malignancies either at diagnosis or during the course of disease were similarly diverse: A/CNNs (18, 32, 48, 77), SVMs (32, 38), K-NN (32, 38), RF (16, 17, 32).

All studies used at least some radiomics features in the models (from a few to several hundred); most studies used automated extraction methods, although some used manual feature extraction (54, 55) and one study included clinical features (15). Only three studies validated model performance on external datasets (34, 35, 55), the remainder choosing cross-validation approaches or a random division of the datasets into training and test sets.

#### 4.2.3 Model discrimination and calibration

AUC values or standard accuracies were provided as the metrics of model performance. No study assessed calibration (i.e., quantifying the uncertainty). With respect to distinguishing gliomas from PCNSL, the AUC values were mainly >0.90, with the occasional study reporting lower values (e.g., 0.85–0.90 and 0.74–0.92 depending on model type in (50) and (42), respectively, and 0.49 and 0.79 independent external datasets in (55) and (34), respectively). Where accuracies were reported, they were similarly usually very high (>90%), except for one study where the maximum accuracy was only 69.2% for the ML approach. Likewise, for those studies discriminating lymphomas from other benign or malignant lesions, the AUC values were generally high (>0.80), except for one attempt to differentiate multiple myeloma from metastases in lumbar vertebrae [best AUC (for a CNN) 0.61 (51)]. Those studies examining disease location or presence reported uniformly good performance (AUCs usually >0.8, accuracies >85%), except for one study identifying patients with and without bone marrow involvement with mantle cell lymphoma, which achieved AUCs up to 0.81 and required the inclusion of laboratory parameters to improve performance (39).

#### 4.2.4 Performance of different ML methods and comparison with radiologist assessment

In studies that compared different ML methods on the same datasets (30, 32, 34, 35, 42, 43, 50, 51, 55), no single approach consistently outperformed the others.

All studies comparing model performance with radiologists or human interpreters reported equivalent (29, 49) or superior (33, 34, 37, 41, 44, 48) performance using ML approaches, except for Swinburne et al. (45), who reported a maximum accuracy of 60.2% for the ML approach to discriminate GBM, PCNSL, and brain metastases with features extracted from routine MRI scans compared with 65.4% and 80.8% for two human readers. However, when the algorithm was added to routine human interpretation, there was a 19% increase in diagnostic yield.

Finally, an SVM-based model (AUC 0.99) compared favorably with blood serum testing to detect patients with bone marrow infiltration with multiple myeloma (38).

#### 4.2.5 Quality assessment

**Figure 2:**
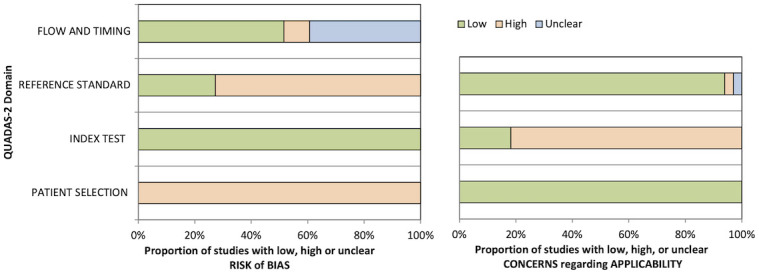
Graphical summary of Quality Assessment of Diagnostic Accuracy Studies 2 (QUADAS-2) results for diagnostic studies.

The quality of diagnostic studies was assessed by QUADAS-2 criteria (**Table 4** and **Figure 2**). In the patient selection risk of bias domain, all studies were considered at a high risk of bias since they needed to be considered as a case–control design because the outcomes were already known before ML algorithms were applied. Conversely, all studies were assessed as a low risk of bias in the index test risk of bias domain because the ground truth was not visible during computational analysis and algorithm development defined a prespecified threshold that was subsequently used in the test phase. In the reference standard risk of bias domain, while nine studies explicitly stated that the reference was interpreted without the knowledge of the ML results, there was uncertainty in the remainder as to whether reference standard interpretation was independent of the index test results. For many of these studies, this lack of information also resulted in uncertainty in the flow and timing domain since the interval between the index and reference tests was uncertain. In the index test domain of concern of applicability, five studies validated the algorithms on external validation cohorts (two *via* a temporal split of the data) and were considered to have a low concern of applicability. All studies were considered to have low concern about applicability in the patient selection domain, and one study had high uncertainty about applicability in the reference standard domain due to the variety of diagnostic techniques used to define the disease status of the cervical nodes assessed in the study (43).

**Table 4:** Quality Assessment of Diagnostic Accuracy Studies 2 (QUADAS-2) assessment for diagnostic studies.

Study	Risk of bias				Applicability concerns		
	Patient selection	Index test	Reference standard	Flow and timing	Patient selection	Index test	Reference standard
Alcaide-Leon et al., 2017 (29)	О	О	?	О	О	?	О
Chen et al., 2020 (30)	О	О	?	?	О	?	О
Chen et al., 2018 (31)	О	О	?	?	О	?	О
Ferjaoui et al., 2021 (32)	О	О	?	?	О	?	О
Hou et al., 2021 (33)	О	О	О	О	О	?	О
Kang et al., 2018 (34)	О	О	О	О	О	О	О
Kim et al., 2018 (35)	О	О	О	О	О	О	О
Kirienko et al., 2020 (14)	О	О	?	О	О	?	О
Kunimatsu et al., 2018 (36)	О	О	?	?	О	О	О
Li et al., 2019 (17)	О	О	?	?	О	О	О
Liu et al., 2012 (37)	О	О	?	?	О	?	О
Martínez-Martínez et al., 2016 (38)	О	О	?	О	О	?	О
Mayerhoefer et al., 2020 (39)	О	О	?	?	О	?	О
McAvoy 2021 (40)	О	О	О	О	О	?	О
Mesguich et al., 2021 (16)	О	О	О	О	О	О	О
Nakagawa et al., 2018 (41)	О	О	?	О	О	?	О
Ou et al., 2019 (15)	О	О	?	О	О	?	О
Priya et al., 2021 (42)	О	О	?	О	О	?	О
Seidler et al., 2019 (43)	О	О	?	О	О	?	О
Sibille et al., 2019 (18)	О	О	?	О	О	?	О
Suh et al., 2018 (44)	О	О	О	О	О	?	О
Swinburne et al., 2019 (45)	О	О	О	О	О	?	О
Tomita et al., 2021 (46)	О	О	О	О	О	?	О
Wang et al., 2021 (47)	О	О	?	О	О	?	О
Wang et al., 2021 (48)	О	О	О	О	О	?	О
Xia et al., 2021 (49)	О	О	?	О	О	?	О
Xiao et al., 2018 (50)	О	О	?	?	О	?	О
Xiong et al., 2021 (51)	О	О	?	?	О	?	О
Xu et al., 2018 (52)	О	О	?	?	О	?	О
Yamasaki et al., 2013 (53)	О	О	?	?	О	?	?
Yang et al., 2017 (54)	О	О	?	О	О	?	О
Yun et al., 2019 (55)	О	О	?	?	О	О	О
Zhang et al., 2021 (56)	О	О	?	?	О	?	О

О low risk, О high risk, ? unclear risk.

^1^ Sample split by time period rather than fully independent.

### 4.3 ML models for segmentation tasks

#### 4.3.1 Applications

A total of 11 studies applied ML to segmentation tasks (**Table 2**) in FDG-PET/CT images: 3 applied it to DLBCL (57, 58, 67), 1 to DLBCL and HL (78), 1 to DLBCL and NHL (64), 1 to natural killer (NK)/T-cell lymphoma (60), 1 to HL (65), and 4 to NHL/lymphoma unspecified (59, 61, 63, 66). One study applied ML to MRI images to segment PCNSL (62).

#### 4.3.2 Model development

CNN-based methods were most commonly applied to segmentation tasks (eight studies (57, 58, 61–65, 67),), but RFs (59), adversarial networks (60), and conditional random fields (66) were also used.

Each study selected a number of different ground truths for comparison with the ML results, including manual selection after 41% SUV_max_ thresholding (57, 58), 41% SUV_max_ thresholding in manually placed ROIs (59, 64, 66), manual segmentation by radiologists but without any further details of the methodology (60–63, 65), or not stated (67).

Models were validated using random splits (60), cross-validation approaches (59, 64, 65, 67), separate (but not fully independent) datasets (57, 61, 63), or validation was unreported or not performed (62, 66).

#### 4.3.3 Model discrimination and calibration

All studies reported Dice similarity coefficients (DSCs). These ranged from 0.71 to 0.95, except for Grossiard et al. (59), which only reported its results descriptively (92% lesion detection, retrieved 75% of tumor volume but overestimated tumor volume by 35%). Calibration (i.e., quantifying the uncertainty) was not assessed in any study.

#### 4.3.4 Quality assessment

**Figure 3:**
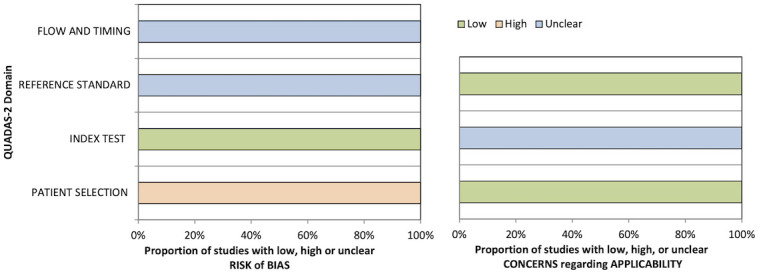
Graphical summary of QUADAS-2 results for segmentation studies.

QUADAS-2 quality assessment results are presented in **Table 5** and **Figure 3**. All studies were considered at a high risk of bias in the patient selection domain and as a low risk of bias in the index test risk of bias domain due to the case–control design and blinded ground truth/prespecified thresholds used, respectively. No study stated whether the reference standard was assessed independent of the index test results or the interval between the index and reference tests; thus, the reference standard and flow/timing biases were deemed unclear. No study validated the proposed ML algorithms on independent cohorts; hence, the concern about the applicability of the index test was deemed uncertain in all cases.

**Table 5:** QUADAS-2 assessment for segmentation studies.

Study	Risk of bias				Applicability concerns		
	Patient selection	Index test	Reference standard	Flow and timing	Patient selection	Index test	Reference standard
Blanc-Durand et al., 2020 (57)	О	О	?	?	О	?	О
Copobianco et al., 2020 (58)	О	О	?	?	О	?	О
Grossiord et al., 2017 (59)	О	О	?	?	О	?	О
Hu et al., 2020 (60)	О	О	?	?	О	?	О
Jemaa et al, 2020 (61)	О	О	?	?	О	?	О
Pennig et al., 2021 (62)	О	О	?	?	О	?	О
Sadik et al., 2018 (63)	О	О	?	?	О	?	О
Weisman et al., 2020 (64)	О	О	?	?	О	?	О
Weisman et al., 2020 (65)	О	О	?	?	О	?	О
Yu et al., 2018 (66)	О	О	?	?	О	?	О
Yuan et al., 2021 (67)	О	О	?	?	О	?	О

О low risk, О high risk, ? unclear risk.

### 4.4 ML models for prognostication or prediction of responses to therapy

#### 4.4.1 Applications

Nine studies applied ML to prognostication or predicting responses to therapy in patients with hematological malignancies (**Table 3**): (i) predicting outcomes (overall survival or progression-free survival) in patients with extranodal NK/T-cell lymphoma, nasal type (69), multiple myeloma (70, 75), DLBCL (71), and mantle cell lymphoma (73) or (ii) predicting responses to therapy in patients with DLBCL (68, 76) and HL (74). One study aimed to identify high-risk cytogenetic (HRC) multiple myeloma patients by applying ML to MRI images (72). Six studies used FDG-PET/CT data, two studies CT data, and one study MRI data.

#### 4.4.2 Model development

Several different ML approaches were applied including a logistic regression classifier (68, 72), random survival forests (RSFs (70);), weakly supervised deep learning (69), ANN/CNNs (71, 73), SVM (72, 74), RF (72, 75, 76), DT (72), K-NN (72), and XGBoost (72). The models used a range of features including not only automatically extracted radiomic features but also clinicopathological variables in two studies (70, 75), laboratory variables in one study (73), and two additional radiological features (nodal site and subjective necrosis) in one study (76). The models were validated by splitting the data or cross-validation, and no study tested the models on independent validation sets.

#### 4.4.3 Model discrimination and calibration

Studies reported model performance with AUCs, hazard ratios (HRs), or prediction errors. The models developed to predict survival outcomes could all discriminate survival differences with the AUCs of 0.83 (73) and 0.88 (test set (69),), the HRs of 4.3 (70) and ~2 (71) between good and poor prognosis groups, or an average prediction error for the PFS of 0.36 (75). With respect to responses to therapy, the reported AUCs for the outcome of interest were between 0.81 and 0.95. Finally, in the study aiming to identify HRC multiple myeloma patients (72), the AUC was 0.84 for the LR model. No study reported calibration statistics.

#### 4.4.4 Quality assessment

Using the NOS, six studies were graded as good, two as “fair”, and one as “poor”, the latter because the cohorts were not comparable because the design or analysis did not adequately control for confounders.

### 4.5 Strengths and limitations of the evaluated studies

This scoping review set out to (i) establish which, if any, ML methods are being used to interpret diagnostic radiology in patients with hematological malignancies; (ii) establish the main applications of ML in hematological cancer radiology; and (iii) identify current research gaps. The review is definitive on the first two aims. Wide, indeed disparate, ML methods have been applied to diagnostic radiology images in patients with hematological malignancies, and there is no consensus on which, if any, approach best suits a particular application. With respect to the second aim, these methods have been applied to three main applications: the diagnosis or discrimination of lymphoma from other disease entities, lesion segmentation to accurately quantify tumor burden in PET-CT images, and for the prognostication or prediction of therapeutic responses.

With three-quarters of ML studies in the hematological oncology radiology arena published in the last 3 years, all the published literature representing preclinical studies, and no ML algorithm yet having entered hematological radiology practice, this scoping review also highlights that the field remains in its infancy. Most of the presented studies, whether evaluated in isolation or compared with radiologist assessment, demonstrate favorable accuracies. Thus, why are these ML algorithms not yet used in clinical practice, and what areas need addressing to facilitate widespread clinical adoption? We propose that three areas must be addressed to progress the clinical application of ML in this field: (i) improvements in model application, validation, comparison, and performance evaluation; (ii) improvements in methodology and reporting standards to reduce bias and promote comparability; and (iii) broadening the study populations to improve generalizability.

#### 4.5.1 Model application, validation, performance evaluation, and comparison

No single ‘best’ ML approach was identified, although some studies compared different ML approaches on the same datasets. Only a few studies validated model accuracy on external datasets, instead splitting the sample or using cross-validation. This limitation is analogous to that seen in molecular biomarker development, where the numerous identified candidate biomarkers for the diagnosis, prognosis, and prediction of responses to therapy do not reach clinical practice because they are not validated in independent datasets (79). Without similar rigorous validation in independent datasets, ML algorithms are likely to be similarly unsuccessful since cross-validation approaches do not account for training dataset bias (e.g., through patient selection or the use of a particular scanner) nor differences in other target populations. While one solution might be to increase the sample size to improve predictive accuracy (79), this does not substitute for applying the same algorithm to fully independent datasets derived from different institutions and geographies. Furthermore, models trained on larger sample sizes do not necessarily perform better (80). It would perhaps be more useful to tailor the sample size to a particular context. In this regard, Riley et al. (81) recently reported a sample size calculation framework for predictive models to help plan the application of ML and avoid underpowered datasets unlikely to yield a meaningful result.

Second, discrimination (i.e., the ability to distinguish a PCNSL from a GBM as measured by the AUC) is not the only metric of model performance, nor is it necessarily the most clinically useful (82). Calibration—that is, establishing the uncertainty of the risk estimates or classification—is also an important performance metric, particularly if intended for clinical use (83). This is most easily understood when ML models are developed to predict the risk of an event, such as relapse: a clinically useful model would not unduly over- or underestimate the risk that a patient will develop a relapse, which might prompt overtreatment (overestimated risk), or, conversely, undertreatment and false reassurance. No study calculated calibration statistics, which is not uncommon in ML; indeed, in one report, 79% of 71 studies using ML for clinical prediction failed to address the calibration problem (84). Given that a highly discriminatory but poorly calibrated model would have poor clinical utility, one solution might be to report probability scores for each outcome (e.g., PCNSL or GBM) along with the calibration statistics of whether the predicted probability scores match the actual probability scores. A metric of the probability that a lesion belongs to a certain class is likely to be much more clinically useful for guiding clinical decision-making based on weak and uncertain binary classifications.

While all diagnostic/segmentation models were developed based on a reference standard (radiologist assessment, either manual or semiautomated), the details of the reference standard were not always clearly reported, leading to large uncertainty in the potential for bias. Furthermore, the *robustness* of the reference standard, i.e., the stability of the diagnosis under varying conditions such as different readers, scanners, or technical protocols, were not reported. This has two main implications; first, a robust standard is essential for the development of an accurate model and therefore predictive performance and (ii) that any developed model may not be generalizable to other settings.

The lack of a standardized reference standard was particularly critical for segmentation tasks, where TMTV was often defined manually without further details of the methodology or using different thresholds. In addition to introducing intra- and interobserver variability and compromising reproducibility, a lack of standardized ground truth makes a meaningful comparison of different studies difficult. While ultimately automated segmentations will eliminate this variability, a methodology for the assessment of tumor burden will need to be standardized to limit error and intercenter variability for clinical development and application. It is currently unclear exactly which ground truth (e.g., SUV ≥41%, SUV ≥2.5, or SUV ≥ mean liver uptake) would be optimal. Further efforts are required to strictly define TMTV, standardize volume segmentation methods, and establish guidelines for the inclusion of tumor-bearing anatomical regions to optimize a completely automated method.

#### 4.5.2 Methodology and reporting standards

Given the high standards of application, validation, evaluation, and comparison required to translate ML algorithms into clinical practice, research must also be conducted and reported to standards that are likely to facilitate this translation. Our quality assessments showed that no study was free of a high risk of bias, particularly due to the process of model development meaning that they were a case–control design; this could at least, in part, be mitigated through a prospective evaluation of independent datasets during validation. Many studies failed to provide adequate details of the reference standard, and few studies were applicable due to the lack of an independent validation step. No study calculated sample sizes *a priori* [for the purposes of model development, as outlined in (81)]. Other important methodological considerations not considered include assessing feature reliability (i.e., that the same features would be extracted under different conditions, such as scans from a different scanner or a different scanning protocol) and full reporting of performance metrics (i.e., a minimum of sensitivity, specificity, positive predictive value, negative predictive value, and the confusion matrix for predictive performance; concordance index and Dice coefficient for survival analysis and segmentation performance, respectively; R squared, mean squared error, root mean squared error, root mean squared logarithmic error, and mean absolute error for regression tasks). For an excellent review of the key considerations when reading and interpreting an ML paper in radiology, see the review by Kocak et al. (85).

Recognizing the importance of clear, transparent, and reproducible scientific communication of the application of ML to medical imaging, the Checklist for Artificial Intelligence in Medical Imaging (CLAIM, first published in 2020) (26) provides a framework to assure high-quality scientific reporting and addresses many of the limitations outlined above. None of the studies reported here—despite many being published within the previous 12 months—used or fully adhered to CLAIM criteria. In a recent systematic review of CLAIM compliance in 186 ML radiology studies, the median CLAIM compliance was 0.40 (IQR 0.33–0.49; calculated as the number of items satisfied over the number of items applicable), suggesting significant room for improvement in the design and reporting of ML studies in radiology; indeed, only 27% documented eligibility criteria and 49% assessed model performance on test data partitions (86). We recommend that all studies use this checklist from the outset.

#### 4.5.3 Study populations

Overall, study populations were small (n<100 for most studies) and heterogeneous (e.g., all grades of lymphomas included). Only two studies were conducted in low- or middle-income countries [LMICs; Turkey and Tunisia (32, 68)]. Developing and validating models in LMICs would have the advantage of improving the generalizability (and therefore utility) of models across the widest range of clinical contexts; second, disparities between models developed in different geographical settings could provide valuable new information about the factors contributing to the variable biology of hematological malignancies. However, we accept that generalizing ML models to LMICs is likely to be challenging since the necessary research infrastructure is often lacking. Nevertheless, given the potential cost benefits of applying AI to resource-poor settings, we believe that generalizing to LMICs is highly desirable and would reduce inequity.

### 4.6 Implications for clinical practice

While the promising performance of the ML models presented in this scoping review provides hope for their future clinical application, clearly, there is still some way to go before they reach clinical “prime-time” for the reasons described above. Given the potential for model overfitting and the lack of independent validation, it is perhaps unsurprising that the headline AUC values for many of the published models are high, and a more realistic appraisal of their clinical benefit will come with time. It is also perhaps worth emphasizing that although comparing ML algorithms with human interpretation is desirable and indeed necessary, an imperfect or inaccurate model does not necessarily imply a lack of clinical value. Ultimately, these ML tools are likely to be best used not to replace radiological assessment but rather to facilitate clinical decision-making, especially for treatment decision-making based on the AI-driven radiological biomarkers of future outcomes or therapeutic responses.

This scoping review highlights that achieving the goal of applying ML to hematological cancer radiology—and ultimately improve clinical outcomes for our patients—will require improvements in study design and clear, transparent, and reproducible reporting. This will not only include reporting diagnostic accuracy or performance but also confirming that it is appropriately calibrated and presented to clinicians in such a way that it can be embedded into clinical practice, for instance, through the use of well-calibrated probability scores. These measures are needed to reduce patient and health system risk, establish trust, and facilitate their widespread adoption. These steps are also needed to facilitate other essential aspects of real-world application and uptake, not least the need to meet regulatory standards. When intended to diagnose, treat, or prevent disease, ML-based software is defined as a medical device under the Food, Drug, and Cosmetic Act (software as a medical device, SaMD). Regulators, including the Food and Drug Administration (FDA), have proposed frameworks for ensuring the safety and efficacy of ML-based SaMDs, which include establishing that the algorithm has a meaningful clinical impact (87). Addressing the research gaps identified in this review would be expected to not only streamline the regulatory process but also improve the quality and applicability of the algorithm in real-world clinical practice.

### 4.7 Study limitations

This study has some limitations. We only searched the PubMed database; thus, papers in other non-biomedical databases may have been missed. We only searched for articles written in English; hence, papers in other languages may not have been included. Although AUC values and Dice scores provide an indication of model discrimination, they are not directly comparable; thus, it is difficult to draw meaningful conclusions about their general applicability to an application of interest, such as discriminating GBM for PCNSL. We identified significant bias and poor or uncertain applicability in nearly every study, and several prognostic/predictive studies similarly failed to control for confounders, which may have also resulted in bias.

## 5 Conclusions

Several research gaps exist and require filling so that robust ML-based models can be used to assist the clinical decision-making of radiologists managing patients with hematological cancers. These include (i) adhering to standardized, high-quality reporting guidelines to reduce bias and improve comparability and generalizability; (ii) validating models in independent cohorts of sufficient size calculated *a priori*; (ii) developing a stricter definition of TMTV and standardizing volume segmentation methods for segmentation tasks; (iv) establishing comprehensive prospective studies that include different tumor grades, comparisons with radiologists, optimal imaging modalities, sequences, and planes; (v) comparing different methods on the same cohort to fully explore and report optimal model generalizability and performance; and (vi) include LMICs in multicentric study designs to further enhance generalizability and reduce inequity. While some of these research gaps are specific to hematological oncology radiology, others—not least establishing and adhering to ML reporting standards, independent validation, and method comparison—are applicable to the application of ML to any diagnostic or predictive task in oncology or hematology, such as predicting outcomes in patients with DLBCL (88). These identified research gaps should help clinicians and computational scientists plan their future research to provide ML-based models that can be applied clinically.

## Data availability statement

The original contributions presented in the study are included in the article/**Supplementary Material**. Further inquiries can be directed to the corresponding author.

## Author contributions

MK and SK had the idea for the article. All authors performed the literature search and wrote parts of the manuscript/assembled the data. SK and MK extracted the data and completed the tables. MK made critical revisions and proofread the manuscript. All authors contributed to the article and approved the submitted version.
